# Appropriateness of recommendations for surveillance colonoscopy after polypectomy – a comparison of adherence to the 2012 and 2020 USMSTF guidelines

**DOI:** 10.21203/rs.3.rs-3870490/v1

**Published:** 2024-01-19

**Authors:** Kacey Idouchi, Mathew J. Gregoski, Don C. Rockey

**Affiliations:** Medical University of South Carolina; Medical University of South Carolina; Medical University of South Carolina

**Keywords:** colon, polyp, cancer, screening, quality, endoscopy, U.S. Multi-Society Task Force

## Abstract

**Background:**

Screening colonoscopy detects precancerous polyps, which when resected, prevents colon cancer. Recommendations for surveillance colonoscopy after polypectomy are based on the U.S. Multi-Society Task Force guidelines (USMSTF).

**Aim:**

to examine provider recommendations based on 2012 and 2020 USMSTF guidelines.

**Methods:**

A prospective analysis was performed to examine provider recommendations for index screening and surveillance colonoscopy from March 2022 to January 2023. Procedures with unknown histology or unsatisfactory bowel preparation were excluded. We recorded polyp morphology, histology, and subsequent recommendations made by endoscopists, to compare to the USMSTF guidelines.

**Results:**

241 patients were included, with 371 endoscopies reviewed. For index screening colonoscopies, 86%, performed between 2012 and 2020, adhered to 2012 guidelines, while 71%, performed after 2020, adhered to the 2020 guidelines. For surveillance colonoscopies, 62% from 2012 and 2020, and 50% after 2020, adhered to the 2012 and 2020 guidelines, respectively (P < 0.001). For polyp types, recommendations after index colonoscopies showed low-risk adenoma (LRA) had 88% adherence to 2012 guidelines versus 73% adherence to 2020 guidelines. For surveillance colonoscopies, LRA had 73% adherence to 2012 guidelines versus 42% adherence to 2020 guidelines (P < 0.001). Recommendations after index colonoscopy showed high-risk adenoma (HRA) had 79% adherence to 2012 guidelines versus 63% adherence to 2020 guidelines. For surveillance colonoscopies, HRA had 88% adherence to the 2012 guidelines versus 69% adherence to 2020 guidelines (P < 0.001).

**Conclusions:**

Adherence declined for the introduction of 2020 guidelines and was poorer after 2nd surveillance exams. Increasing the evidence for interval recommendations may increase guideline adherence.

## Introduction

Colorectal carcinoma (CRC) is the second leading cause of cancer death in the US [1]. However, because pre-malignant lesions such as adenomas take years to develop into a malignant lesion, screening colonoscopies have been effective in reducing mortality from CRC [2–4]. Thus, colonoscopies have been the gold standard for CRC screening. During a colonoscopy, any precancerous polyps found are removed, and endoscopists make follow-up recommendations for their surveillance colonoscopy. U.S. Multi-Society Task Force (USMSTF) guide most of these recommendations, and update their guidelines based on new data. For example, in 2021 recommendations from the USMSTF suggest that colorectal cancer screening in average-risk individuals should start at age 45, instead of 50 [3,5,6]. A major component of the USMSTF guidelines is to recommend surveillance intervals that balance between precancerous polyp detection and over-scoping patients [7,8].

The USMSTF provide recommendations based on poly types. Polyps are categorized based on histology, number, location, and size, to assess the risk of it developing into CRC. Polyps of interest include, but are not limited to, adenomas, sessile serrated adenoma/polyp or sessile serrated lesions, hyperplastic polyps, and traditional serrated adenomas [9,10]. Importantly, after identification and categorization of the polyps, evidence-based recommendations given by the USMSTF for surveillance colonoscopy suggest that these exams typically occur between 3 and 10 years after the previous exam (**Supplemental Tables 1 and 2**) [3,11].

Despite the widespread availability of specific guidelines, which inform the appropriate interval after previous polypectomy, it has been our observation that surveillance colonoscopy is not always performed according to USMSTF guidelines. Thus, to better understand the current practice around surveillance colonoscopy, we examined provider recommendations for follow-up colonoscopy – including in comparison to USMSTF guidelines available at the time the colonoscopy was performed (i.e., 2012 and 2020).

## Methods

### Study Population and Design

This was a prospective observational analysis of intervals for colonoscopy after index screening colonoscopy or after a surveillance colonoscopy. The study was conducted at a tertiary care academic medical center from March 2022 to January 2023. We included average-risk patients aged ≥ 50 years for the 2012 guidelines and ≥ 45 years after 2021 to account for the change in recommended age for CRC screening. Any patient having an inadequate bowel preparation (i.e., other than “adequate”, “good”, or “excellent”), unknown histology of previous polyps, or who were over the age of 70 were excluded. Patients with a history of inflammatory bowel disease, CRC, partial polypectomy, familial adenomatous polyposis, Lynch syndrome, MYH-associated polyposis, or juvenile polyposis were also excluded. We excluded colonoscopy reports that had multiple types of polyps as the old and new guidelines do not address the intervals for multiple polyp types. The interval in which colonoscopy was performed was compared to the 2012 or 2020 USMSTF guidelines - depending on when the index colonoscopy was performed (**Supplemental Table 1,2,3**).

We examined both index screening colonoscopy as well as surveillance colonoscopies that were done by gastroenterologists. First-time screening colonoscopies after 2012 and 2020 were obtained for their respective guidelines. Patients having a surveillance colonoscopy between March 2022 and January 2023 were assessed based on the 2020 USMSTF guidelines. Additionally, since they had a previous index screening colonoscopy, or surveillance colonoscopy, we examined recommendations following that examination - compared with 2012 USMSTF guidelines. This study was approved by the Medical University of South Carolina Institutional Review Board (protocol no. Pro00116200).

### Statistical analysis

An a priori power analyses was conducted to identify an appropriate sample size for the study. Given that adherence to time appropriate USMSTF recommendations for followup should be 100%, but recognizing that this would be unlikely in practice, we judged that an expected adherence to time appropriate USMSTF recommendations of 80% would not be unexpected. Hover, based on previous data demonstrating that the adherence rate to followup colonoscopy of 49% [12], we estimated that the recommendation for follow-up might likewise be similar in our cohort. Therefore, using these data to inform expected and actual adherence to time appropriate USMSTF recommendations, for independent proportions, with a significance of α = 0.05 and power = 0.8, a minimum sample size of 39 was needed for each category, to detect an estimated difference between expected adherence of at least 80% and actual adherence of 50%.

Statistical analyses were performed using SPSS version 28 (IBM corporation, Armonk, NY). Descriptive data are presented as percentages and numbers. Categorical variables were describe using frequency and percentages and were analyzed using X^2^ analyses or Fisher’s exact tests as appropriate. Statistical significance was defined as a *p* value of less than 0.05. Analyses including more than 2 subgroups utilized Bonferroni adjusted p-values for multiple comparisons.

### Determining compliance

For each patient we collected the following data: age, sex, race, polyp morphology, polyp size, polyp location, number of polyps, quality of bowel preparation, pathology report, dates of each colonoscopy and gastroenterologist recommendation. We determined, based on polyp pathology what the appropriate recommendations were with the 2012 and 2020 USMSTF guidelines. USMSTF recommendations were then compared to formally recorded recommendations (i.e., in the medical record), after endoscopist review of the formal pathology report.

## Results

**Figure 1:**
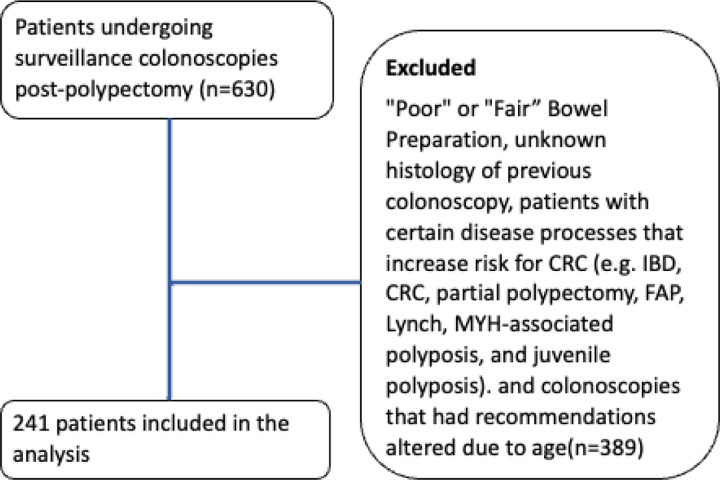
Patients. Shown is a consort diagram of patients included in the study.

Of the 630 patients evaluated, 241 patients had surveillance colonoscopies and were included in the study (Fig. 1). Overall, 200 colonoscopies were analyzed between 2012 and 2020 and 171 colonoscopies were analyzed after 2020 with a total of 371 colonoscopy recommendations analyzed. 19 endoscopists were evaluated. All included patients had “good”, “excellent”, or “adequate” bowel preparation and had the polyp histology of their previous colonoscopy documented. The average age of patients was 63 ± 8years, with 55% being female, 67% Caucasian, 31% African American, and 2% Asian (Table 1).

In aggregate, index screening and surveillance colonoscopies performed between 2012 and 2020 had an adherence rate of 153/200 (76%) to USMSTF guidelines for recommended interval follow-up colonoscopy (Table 2). When combined, index screening and surveillance colonoscopies performed after 2020 had an adherence rate of 93/171 (54%) USMSTF guidelines for recommended interval follow-up colonoscopy (p < 0.001 for the difference in adherence to recommended 2012 and 2020 guidelines).

Of the 118 patients had an initial index screening colonoscopy performed between 2012 and 2020, and 102 (86%) adhered to the 2012 USMSTF guidelines for the recommended interval for surveillance colonoscopy (Table 3a and 3b). For the 2020 time period, there were 43 initial index screening colonoscopies performed and 31 (72%) had recommendations which adhered to the 2020 USMSTF guidelines for the recommended interval follow-up colonoscopy (p = 0.02 for the difference in adherence to recommended 2012 and 2020 guidelines).

A total of 69 patients underwent surveillance colonoscopy between 2012 and 2020, and 43 (62%) adhered to the 2012 USMSTF guidelines for recommended interval follow-up colonoscopy (Table 3a and 3b). For the 2020 time period, there were 103 surveillance colonoscopies performed, and 52 (50%) had recommendations that adhered to the 2020 USMSTF guidelines for the recommended interval follow-up colonoscopy.

Interestingly, there was a difference in adherence to 2012 published guidelines when comparing recommendations after index colonoscopy to recommendations after the 1st surveillance colonoscopy was performed (102/118 (86%) vs. 43/69 (62%), p < 0.001, Table 3a). Similar finding were identified after publication of 2020 guidelines (31/43 (72%) vs. 52/103 (50%), p = < 0.001 Table 3b).

Thirteen patients that underwent a second surveillance colonoscopy between 2012 and 2020, and 8 (62%) adhered to the 2012 USMSTF guidelines for recommended interval follow-up colonoscopy (Table 3a and 3b). For the 2020 time period, there were 25 second surveillance colonoscopies performed, and 10 (40%) had recommendations that adhered to the 2020 USMSTF guidelines for recommended interval follow-up.

Of repeat colonoscopies that did not adhere to the USMSTF guidelines for surveillance intervals, 97% were performed too early. There was also a progressive decline in adherence rate for recommendations after the index colonoscopy and after the 1st colonoscopy that had followed the 2012 USMSTF guidelines, with an adherence rate declining from 86–62%. With the 2020 USMSTF guidelines, the adherence rate declined from 72–50%.(Table 3a and 3b).

An analysis of adherence rate based on adenoma features (i.e., low-risk adenomas (LRA) and high-risk adenomas (HRA)) was also performed (Table 4). The adherence rate with the 2012 USMSTF guidelines, after the index colonoscopy, was 71/81 (88%) for LRA and 41/52 (79%) for HRA (P < 0.001). The adherence rate with the 2012 USMSTF guidelines, after the 1st surveillance was 19/26 (73%) for LRAs and 22/25 (88%) for HRAs (P < 0.001). The adherence rate with the 2020 USMSTF guidelines, after the index colonoscopy was 33/45 (73%) for LRAs and 12/19 (63%) for HRAs (P < 0.001). The adherence rate with the 2020 USMSTF guidelines, after the 1st surveillance was 21/50 (42%) for LRAs and 27/48 (69%) for HRAs (P < 0.001).

Polyps such as sessile serrated polyps (SSPs) were not analyzed as there was not a large enough sample size to detect predicted differences in adherence rates as highlighted in Methods. Hyperplastic polyps (HPs) were also commonly found alongside LRA and HRA, thus making it difficult analyze recommendations for isolated HPs. Thus, HPs were not analyzed in this study.

## Discussion

Current evidence indicates that index screening and surveillance for colon cancer is effective [3,4,13]. However, specifics around appropriate and proper surveillance colonoscopy after identification of polyps is challenging and perhaps controversial [11,14] because recommendations should in theory balance the benefit of identifying early malignancy vs. harm engendered by the risks associated with colonoscopy [15]. For example, recommending inappropriate intervals for surveillance colonoscopies increases healthcare costs, increases the usage of medical resources, and decreases the capacity for patients who are in greater need of screening or surveillance colonoscopies [16,17]. Performing colonoscopy more frequently than needed also means an increased risk for complications such as bleeding or perforation [15–17].

Our data suggests that recommended surveillance colonoscopy intervals adhere more closely to the 2012 than the 2020 USMSTF guidelines for interval colonoscopy follow-up. Notably, recommended intervals for the 1st surveillance after index screening colonoscopy had a much lower adherence rate overall, regardless of the time frame for USMSTF guidelines. Finally, 2nd surveillance intervals were less closely followed than the 1st surveillance interval and were worse for the 2020 than the 2012 guidelines. Interestingly, adherence rates appeared to follow these trends, regardless of polyp features (i.e., whether LRA or HRA).

The 2020 guidelines had some important updates and changes compared to the 2012 guidelines. LRAs had the lowest adherence rate, which could be explained by the notable recommendation changes as follows: for 1 to 2 tubular adenomas < 10mm, the 2012 recommendation for follow-up was in 5–10 years, which was moved to 7–10 years with the new guidelines (**Supplemental table 2**). The dip in LRA compliance could potentially be explained by the change in the 2020 guidelines for the recommended interval for followup of 1–2 tubular adenomas (i.e., increased to 7–10 years; that is to say that with the new guidelines, the new minimum recommended interval had increased by 2 years. The new guidelines are also further stratified with follow-up recommendations for tubular adenomas after the 1st surveillance is performed. In contrast, the 2012 guidelines did not incorporate findings from previous colonoscopies in recommended intervals. With only “moderate” quality of evidence for follow-up of tubular adenomas, endoscopists may not be comfortable in recommending a longer waiting for the next surveillance colonoscopy [11]. This could be consistent with the stated “very low”, “weak”, or “moderate” evidence supporting some of the USMSTF guidelines [11]. In a study that surveyed physician opinions about 2012 polyp surveillance guidelines, 57% of gastroenterologists found the guidelines to be “very influential” in their practice. They also reported that although gastroenterologists were familiar with the guidelines, 76% disagreed with the recommendations [18].

Other theories of low compliance has been suggested, such as lack of guideline awareness, but a recent study suggested that this may not explain the low compliance [19]. A study showed that when endoscopists were given LRA and HRA surveys with clinical vignettes, they were able to answer them correctly despite their low compliance in clinical practice [19]. Again, endoscopists may feel the low strength of evidence may not be justifiable enough to follow it in practice. This study was limited as it only accounted for screening colonoscopies and did not examine surveillance exams and was only performed at one medical center and thus may not be generalizable. Previous studies have examined colonoscopy surveillance adherence. In a 2019 meta-analysis that examined rates of adherence to surveillance guidelines (2012) based on physician recommendations in 16 studies found that the appropriate adherence rate for colonoscopy surveillance adherence rate was 49%, with a range of 15–91% [12]. Some studies have proposed that (poor) quality of bowel preparation and concern about missed lesions or unclear histological findings of polyps, may possibly explain the shorter interval recommendations that have been observed [20,21].

We also have considered the possibility that the colonoscopies examined were during the COVID-19 pandemic, which may have affected follow-up recommendations. A systematic review showed that COVID-19 had an impact on the number of screening colonoscopies that performed during the pandemic [22], revealing that the number of colonoscopies decreased. Whether this could have had an effect on follow-up recommendations is unclear, though we speculate that the impact of COVID-19 on performance of colonoscopy would be unlikely to have a bearing on recommendations for future colonoscopies. Interestingly, consistent with our findings, a study that set out to determine whether the volume of colonoscopy could be reasonably reduced by more rigorously implementing 2020 USPSTF guidelines to help alleviate the downstream effort of COVID-19, found that 15–21% of colonoscopies at their institution qualified to be rescheduled to a future year [23].

We recognize strengths and limitations of our study. A critical strength of the study was that we performed a power analysis a priori to ensure an adequate cohort of patients in each of the 2012 and 2020 cohorts. We also examined adherence according to adenoma features (i.e., LRA and HRA), and we took care to exclude patients with inadequate preparations or pathology reports. In terms of weaknesses, perhaps the most important weakness was that this study was performed at a single center, and the practice at one institution may be different than at other institutions. However, we speculate that given current national healthcare policies, the practice at our institution is likely similar to others. We did not include patients in whom surveillance was recommended past the age of 75 and therefore cannot comment on practices in this age group. It should be emphasized that this was intentional since guidelines for surveillance past the age of 75 are controversial, and guidance is limited [24]. Further, the US Preventive Services Task Force recommends CRC screenings until age 75 and recommends individualized screening decisions for adults aged 76 to 85 years of age [5,24].

In conclusion, our findings indicate that adherence to surveillance guideline recommendations after index screening was better than surveillance colonoscopy and that adherence to surveillance guideline recommendations was especially poor after 2nd surveillance exams. Further, adherence has declined after the introduction of 2020 guidelines, even while it has been published for the past 2–3 years. We speculate the 2012 USMSTF guidelines may be easier to follow than the updated guidelines, but that further education and perhaps stronger evidence may help improved adherence to 2020 USMSTF guidelines.

## Figures and Tables

**Table 1:** Demographic data

Demographic (n = 241)	Total (%) or Mean (± SD)
**Race**	
Caucasian	162 (67%)
African American	76 (31%)
Hispanic/Latino	0 (0%)
Asian	3 (2%)
**Gender**	
Female	133(55%)
**Age**	
Age (Std Error)	63 ± 8

**Table 2:** Adherence to recommended 2012 and 2020 guidelines (all index and surveillance recommendations)

	All colonoscopies performed between 2012 and 2020 that followed the 2012 guidelines	All colonoscopies performed after 2020 that followed the 2020 guidelines	P-value
Compliant	153 (76%)	93 (54%)	<0.001
Non-compliant	47 (24%)	78 (46%)	
Total	200	171	

**Table 3:** **a.** 2012 guideline adherence rate of surveillance colonoscopies

	Followed 2012 guidelines for recommendations after the index colonoscopy was performed between 2012 and 2020	Followed 2012 guidelines for recommendations after the 1st surveillance colonoscopy was performed between 2012 and 2020	Followed 2012 guidelines for recommendations after the 2nd surveillance colonoscopy was performed between 2012 and 2020	P-value
Adherent	102 (86%)	43 (62%)	8 (62%)	<0.001
Non-adherent	16 (14%)	26 (38%)	5 (38%)	
Total	118	69	13	

**Table 3:** **b.** 2020 guideline adherence rate of surveillance colonoscopies

	Followed 2020 guidelines for recommendations after the index colonoscopy was performed after 2020	Followed 2020 guidelines for recommendations after the 1st surveillance colonoscopy was performed after 2020	Followed 2020 guidelines for recommendations after the 2nd surveillance colonoscopy was performed after 2020	P-value
Adherent	31 (72%)	52 (50%)	10 (40%)	<0.001
Non-adherent	12 (28%)	51 (50%)	15 (60%)	
Total	43	103	25	

**Table 4:** a. 2012 guideline adherence rates in low-risk and high-risk adenomas for index and surveillance colonoscopies

	Index		1st Surveillance		P-value
	Adherent	Non-adherent	Adherent	Non-adherent	<0.001
Low-risk adenoma	71 (88%)	10 (12%)	19 (73%)	7 (27%)	
High-risk adenoma	41 (79%)	11 (21%)	22 (88%)	3 (12%)	
Total polyps	112	22	41	10	

**Table 4:** b. 2020 guideline adherence rates in low-risk and high-risk adenoma for index and surveillance colonoscopies

	Index		1st Surveillance		P-value
	Adherent	Non-adherent	Adherent	Non-adherent	<0.001
Low-risk adenoma	33 (73%)	12 (27%)	21 (42%)	29 (58%)	
High-risk adenoma	12 (63%)	7 (37%)	27 (69%)	12 (31%)	
Total polyps	45	19	48	41	

## Abbreviations

CRC: colorectal Cancer
HRA: high–risk adenoma
LRA: low–risk adenoma
USMSTF: U.S. Multi–Society Task Force guidelines
SSP: sessile serrated polyp
HP: hyperplastic polyp
